# A prospective study of *Helicobacter pylori *in relation to the risk for pancreatic cancer

**DOI:** 10.1186/1471-2407-8-321

**Published:** 2008-11-05

**Authors:** Björn Lindkvist, Dorthe Johansen, Anders Borgström, Jonas Manjer

**Affiliations:** 1 Institute of Medicine, Sahlgren's Academy, University of Göteborg, Gothenburg, Sweden; 2 Department of Clinical Sciences, Malmö University Hospital, Lund University, Malmö, Sweden; 3 The Malmö Diet and Cancer Study, Malmö University Hospital, Malmö, Sweden; 4 Department of Internal Medicine, Division of Gastroenterology and Hepatology, Med pol II, Sahlgrenska University Hospital, SE-413 45 Gothenburg, Sweden

## Abstract

**Background:**

The relationship between *Helicobacter pylori *infection and pancreatic cancer has been investigated in three previous studies with contradictory results. The aim of the present study was to investigate the association between *H. pylori *seropositivity and the risk for pancreatic cancer in a nested case-control study within a population based cohort.

**Methods:**

Selected birth-year cohorts (born 1921–1949) of residents in Malmö, Sweden, were invited to a health screening investigation. A total of 33 346 subjects participated. Cases with pancreatic cancer (n = 87) were matched to controls (n = 263) using age, sex and time for baseline investigation as matching variables. *H. pylori *serology was analysed in stored serum samples using an enzyme-linked immunosorbent assay. Odds ratios (OR) for pancreatic cancer were calculated with 95% confidence intervals (CI) using logistic regression.

**Results:**

*H. pylori *seropositivity was not associated with pancreatic cancer in the total cohort (adjusted OR 1.25 (0.75–2.09)). However, a statistically significant association was found in never smokers (OR 3.81 (1.06–13.63) adjusted for alcohol consumption) and a borderline statistically significant association was found in subjects with low alcohol consumption (OR 2.13 (0.97–4.69) adjusted for smoking).

**Conclusion:**

We conclude that no association between *H. pylori *infection and the risk for pancreatic cancer was found in the total cohort. However, in never smokers and in subjects with low risk alcohol consumption, a positive *H. pylori *serology was associated with an increased risk for pancreatic cancer. These findings should be interpreted cautiously due to the limited number of cases in these subgroups.

## Background

Pancreatic cancer is a relatively infrequent form of cancer but due to the poor prognosis associated with the disease, it ranks eight among the leading causes of cancer related deaths worldwide [1]. Smoking is the most well documented risk factors for pancreatic cancer, estimated to account for about 25% of all cases [2]. Alcohol consumption is not an established risk factor for pancreatic cancer, but there is a well known association between alcohol consumption and chronic pancreatitis, and chronic pancreatitis is associated with an increased risk for pancreatic cancer [3].

*Helicobacter pylori *infection has previously been associated with gastric cancer [4-7] and mucosa-associated lymphoid tissue lymphoma [8,9]. The association between *H. pylori *infection and pancreatic cancer has been investigated in three previous studies. One case-control study and one prospective cohort study among smoking men have both indicated an about doubled risk for pancreatic cancer in *H. pylori *infected individuals [10,11]. However, this association could not be confirmed in a recent nested case-control study performed in a cohort of subscribers to a medical care program in the US [12].

The Malmö Preventive Project was set up in 1974 with the main purpose to screen the middle-aged population for cardiovascular disease risk factors [13]. The cohort consists of 33 346 individuals subjected to a health screening investigation sometime between 1974 and 1992 including physical examination and a self-administered questionnaire. Stored blood samples are available from the baseline investigation.

The objective of the present study was to investigate the association between *H. pylori *infection and the risk of pancreatic adenocarcinoma in relation to smoking and drinking habits in this population based cohort.

## Methods

### The Malmö Preventive Project Cohort

In 1974, a Department of Preventive Medicine was set up within The Department of Medicine at Malmö University Hospital [13]. The main goal was to screen the middle-aged population for risk factors for cardiovascular diseases, diabetes mellitus and alcoholism. Complete birth-year cohorts of registered residents in Malmö, Sweden, were invited by letter to a health screening investigation from 1974 to 1992. All men born in 1921, 1926–1942, 1944, 1946 and in 1948–49, and all women born in 1926, 1928, 1930–1938, 1941 and in 1949, were included. The attendance rate was high (71%), and when the recruitment ended a total of 33 346 individuals (22 444 men and 10 902 women) had participated. At baseline examination subjects responded to a self-administered questionnaire, weight and height were measured and blood samples were collected. Selected biochemical analyses were performed at baseline and the remaining biological material was stored in a biological specimen bank at -20°C.

#### Baseline exposure assessment

Weight and height were measured at baseline investigation by a trained nurse. Body mass index (BMI) was calculated as weight (kg) divided by length (m)^2^. Smoking habits were assessed by questionnaire at baseline investigation. The question "Have you ever been smoking on a daily basis for at least six months?" was used to separate those who had ever smoked ("ever smokers") from those who had never smoked ("never smokers"). Ever smokers were classified as current smokers if they had confirmed current smoking, the remaining were classified as former smokers. This procedure has been described in detail previously [14]. Alcohol consumption was estimated using a scoring system that has been described in a previous publication[14] based on a modified version of the Michigan Alcoholism Screening Test [15], the "Malmö modification of the brief MAST" (Mm-MAST)[16]. In brief, subjects answered 7 yes or no questions regarding drinking habits, these answers were integrated into a scoring system which was used to classify alcohol consumption as "low" "intermediate" or "high" risk consumption [14]. No attempts to quantify alcohol consumption were made. Gamma glutamyl transferase (γ-GT) was measured at the time for baseline investigation using γ-glutamyl-p-nitroanilin as a substrate [17]. Values of γ-GT were available for all but 2 subjects. γ-GT values were used as an alternative marker for alcohol consumption. The cohort was divided into tertiles of γ-GT values, cut-off levels for the different tertiles were identified in cases in order to construct three groups of individuals with an equal number of cases in each group.

### Identification of cases and matching of control subjects

Cases of pancreatic cancer were identified by record linkage of the Malmö Preventive Project cohort database to the Swedish Cancer Registry using the ICD 7 diagnostic code for pancreatic cancer (157). Cases that occurred until 31 December 1999 were included in the study. The record linkage yielded 117 subjects registered with the diagnosis of pancreatic cancer in the Malmö Preventive Project. Clinical and pathology records were reviewed in all subjects. The diagnosis of pancreatic adenocarcinoma could be verified in 113 cases. Four cases were found to have been erroneously registered as pancreatic adenocarcinomas (two islet cell tumors, one endocrine tumor and one anaplastic malignancy) and were therefore excluded from further analysis. All 113 cases were matched to 3 controls resulting in a set of 452 subjects. Age was matched as +/- 180 days and time of baseline examination as +/- 180 days. A large proportion of subjects examined during the first year (Oct. 1974–1975) had no available biological material. It was therefore decided that only subjects examined from 1 Jan. 1976 should be included in analyses, resulting in a data set of 104 cases and 311 controls. Following sample retrieval and aliquoting, 87 cases had the necessary amount of biological material. Considering the relatively large number of subjects with missing biological material, the matched analysis was abandoned at this point. The laboratory analyses were, hence, performed in 87 risk sets ordered as 3 controls and 1 case. Following analysis of another 3 controls, no more cases were available and the laboratory analyses were finished. One analyzed control subject was excluded because of failure in the *H. pylori *serology analysis. In all, 350 subjects were included in the data analyses; 87 cases and 263 controls, excluding 102 of the 452 subjects initially intended for the study. Age, BMI and smoking habits were similar in excluded and included subjects but there was a higher proportion of men and a higher proportion of missing information on alcohol consumption in the excluded group. This difference was expected since mostly men were investigated during the early phase of the study and questions on alcohol were not introduced in the questionnaire until December 1976. Among included cases, evidence for the diagnosis of pancreatic adenocarcinoma was found in pancreatic resection specimens in nine cases, at autopsy in 40 cases and by fine needle biopsy in 36 cases (in 31 cases the biopsy was conclusive for pancreatic adenocarcinoma and in 5 cases the biopsy showed low differentiated adenocarcinoma of unclear origin but radiological findings were indicative for a primary pancreatic tumor). In the remaining two cases the diagnosis was based on the clinical picture and radiological findings without any cytological or histopathological data.

### *H. pylori *serology

IgG antibodies against *H. pylori *were measured by an in house enzyme-linked immunosorbent assay (ELISA) at the department of microbiology, Malmö University Hospital, in blood samples from cases and controls stored at baseline investigation [7]. Absorbance > 0.70 was regarded as a positive test. The validity of this assay has previously been investigated in a similar setting on stored blood samples from the same cohort. Immunoreactivity was found to be stable over time in that study. A bimodal distribution in the absorbance level was demonstrated with two distinct populations well separated by the cut-off level of 0.70 [7].

### Statistical analysis

All statistical calculations were performed using the software SPSS 15.0. Median age, body mass index and time from baseline investigation to analysis at baseline investigation were calculated. The distribution of baseline characteristics was compared between cases and controls and between subjects with a positive and a negative *H. pylori *serology. Unconditional logistic regression was used to estimate crude and adjusted odds ratios (OR) with a 95% confidence interval (CI). Conditional logistic regression was considered inappropriate since the case-control matching was disrupted due to the fact that blood samples were missing for several cases and controls. Adjusted OR were obtained by including age, sex, body mass index (BMI), smoking, alcohol consumption according to the Mm-MAST test, *H. pylori *serology and time from baseline investigation to analysis, in the logistic regression model. Simultaneous adjustment for Mm-MAST and γ-GT tertiles was considered inappropriate since both are used as surrogate markers for the same parameter, ie alcohol consumption. Mm-MAST score was chosen as the principal marker for alcohol abuse, since this variable was considered to be a more specific marker for alcohol abuse than γ-GT. However, all calculations were repeated replacing Mm-MAST category with γ-GT tertiles for comparison. OR for pancreatic cancer in relation to *H. pylori *serology was further studied in separate strata of smoking habits, Mm-MAST category, γ-GT tertiles and BMI. Due to the small number of cases in each stratum of smoking status or alcohol consumption, the number of covariates included had to be reduced in these analyses. Possible covariates (age, sex, body mass index (BMI), smoking, alcohol consumption measured by Mm-MAST category, and time from baseline investigation to analysis) were therefore included one at a time in order to determine factors with a significant impact on the association between *H. pylori *serology and pancreatic cancer. As a comparison to the overall risk calculations, OR adjusted for all covariates were calculated in order to allow assessment of the stability of the statistical model, although the number of entered covariates in this final analysis was formally unduly large.

### Ethical Approval

The Ethical Committee at Lund University approved the current study (LU 828-02; 6 Feb. 2003). In line with the requirements of the local ethical committee, all participants in the Malmö Preventive Project were informed of the present study by advertisements in local newspapers. The possibility to withdraw from the current analysis was explicitly stated.

## Results

### Baseline characteristics

Baseline characteristics for included cases and controls are presented in table 1. Cases and controls were highly similar regarding matching factors despite the fact that the case-control matching was partially disrupted (due to missing blood samples as stated above). There was a higher proportion of current smokers among cases. Alcohol consumption, as measured by the Mm-MAST test, was similar in cases and controls. The median age for diagnosis among cases was 60.7 years (range 47.6–76.5). Baseline characteristics by *H. pylori *serology are presented in table 2. There was a slightly higher proportion of current smokers among *H. pylori *positive subjects. Groups were highly similar with regards to age, sex distribution and time from baseline investigation to analysis of *H. pylori *serology.

**Table 1:** Baseline characteristics of case with pancreatic cancer and control subjects

Factor		Cases (n = 87)	Controls (n = 263)	p-value
Age (years)		*47.9* *(37.7–60.6)*	*47.5* *(37.3–60.6)*	0.53*
Sex	Male	58 (66.7%)	187 (71.1%)	
	Female	29 (33.3%)	76 (28.9%)	
Time from baseline investigation to analysis (years)		*24.8* *(14.3–28.8)*	*25.1* *(18.1–29.0)*	0.14*
Body mass index (kg/m^2^)		*23.8* *(18.0–41.0)*	*24.2* *(17.6–34.2)*	0.82*
Smoking status	Never smoker	13 (14.9%)	88 (33.5%)	0.002**
	Current smoker	55 (63.2%)	115 (43.7%)	
	Former	19 (21.8%)	60 (22.8%)	
Mm-MAST category†	Low	35 (40.2%)	129 (49.0%)	0.48**
	Intermediate	42 (48.3%)	113 (43.0%)	
	High	7 (8.0%)	14 (5.3%)	
	Missing	3 (3.4%)	7 (2.7%)	
γ-glutamyle transferase-tertiles (μkat/l)	1 (< 0.35)	31 (35.6%)	98 (37.5%)	0.63**
	2 (0.35–0.60)	27 (31.0%)	90 (34.5%)	
	3(> 0.60)	29 (33.3%)	73 (28.0%)	
*Helicobacter pyori *serology	Negative	48 (55.2%)	163 (62.0%)	0.26**
	Positive	39 (44.8%)	100 (38.0%)	

Numbers represent n with column percent in brackets except for numbers in italics which represent medians with range in brackets.

*Mann-Whitney U-test ** Chi-square test.

†Mm-MAST, Malmö modification of the brief Michigan Alcoholism Screening Test.

**Table 2:** Baseline characteristics by *Helicobacter pylori *serology

		*H. pylori *serology		
			
Factor	Category	Negative	Positive	p-value
Age (years)		*47.6* *(37.3–60.6)*	*47.4* *(37.7–60.6)*	0.53*
Sex	Male	144 (68.2%)	101 (72.7%)	0.38**
	Female	67 (31.8%)	38 (27.3%)	
Time from baseline investigation to analysis (years)		*25.1* *(14.3–28.9)*	*24.8* *(14.3–29.0)*	0.14*
Body mass index (kg/m^2^)		*24.0* *(17.6–37.8)*	*24.2* *(18.2–41.0)*	0.82*
Smoking status	Never smoker	69 (32.7%)	32 (23.0%)	0.14**
	Current smoker	96 (45.5%)	74 (53.2%)	
	Former	46 (21.8%)	33 (23.7%)	
Mm-MAST category†	Low	97 (46.0%)	67 (48.2%)	0.81**
	Intermediate	97 (46.0%)	58 (41.7%)	
	High	12 (5.7%)	9 (6.5%)	
	Missing	5 (2.4%)	5 (3.6%)	
γ-glutamyle transferase-tertiles (μkat/l)	1 (< 0.35)	87 (41.2%)	42 (30.7%)	0.14**
	2 (0.35–0.60)	66 (31.3%)	51 (37.2%)	
	3(> 0.60)	58 (27.5%)	44 (32.1%)	

Numbers represent n with column percent in bracket except for numbers in italics which represent medians with range in brackets.

*Mann-Whitney U-test ** Chi-square test.

†Mm-MAST, Malmö modification of the brief Michigan Alcoholism Screening Test.

### Association between *H. pylori *serology and pancreatic cancer in the total cohort

The risk for pancreatic cancer in different categories of *H. pylori *serology, smoking habits and alcohol consumption are presented in table 3 as crude OR and OR adjusted for age, sex, BMI, *H. pylori *serology, smoking status, Mm-MAST category and time from baseline investigation to analysis. There was no association between *H. pylori *seropositivity and pancreatic cancer in the overall analysis (adjusted OR = 1.25 (0.75–2.09)). Current smoking was associated with a statistically significantly increased OR for pancreatic cancer (adjusted OR = 3.59 (1.79–7.21)), and there was a borderline significant increase in the OR for pancreatic cancer among former smokers (adjusted OR = 2.16 (0.97–4.82)). A tendency towards increased OR's for pancreatic cancer in subjects with intermediate and high risk alcohol consumption was observed but the association was not statistically significant in any of these groups (adjusted OR = 1.33 (0.77–2.32) and 1.84 (0.65–5.23), respectively).

**Table 3:** Crude and adjusted odds ratios (OR) for pancreatic cancer with 95% confidence intervals (CI) by *Helicobacter pylori *serology, smoking status and alcohol consumption

Factor	Status	Crude OR (95% CI)	OR (95% CI)*
*H. pylori *serology	Negative	1.00 (reference)	1.00 (reference)
	Positive	1.32 (0.81–2.16)	1.25 (0.75–2.09)
			
Smoking	Never smoker	1.00 (reference)	1.00 (reference)
	Current smoker	3.24 (1.67–6.30)	3.59 (1.79–7.21)
	Former smoker	2.14 (0.99–4.67)	2.16 (0.97–4.82)
			
Mm-MAST** category	Low	1.00 (reference)	1.00 (reference)
	Intermediate	1.37 (0.82–2.29)	1.33 (0.77–2.32)
	High	1.84 (0.69–4.92)	1.84 (0.65–5.23)
	Missing	1.58 (0.39–6.43)	1.86 (0.42–8.15)
			
γ-glutamyle transferase-tertiles (μkat/l)	1 (< 0.35)	1.00 (reference)	1.00 (reference)
	2 (0.35–0.60)	0.95 (0.53–1.71)	0.83 (0.44–1.59)
	3 (> 0.60)	1.27 (0.70–2.27)	1.07 (0.54–2.12)

* Odds ratios for the risk factors *H. pylori *serology, smoking and Mm-MAST category are adjusted for age, sex, body mass index, *H. pylori *serology status, smoking status, time from baseline investigation to analysis and alcohol consumption. Mm-MAST is used as surrogate marker for alcohol consumption, no adjustment is done for γ-glutamyle transferase values. Adjusted OR for γ-glutamyle transferase-tertiles are adjusted for the same cofactors except for Mm-MAST category.

** Mm-MAST, Malmö modification of the brief Michigan Alcoholism Screening Test.

### Stratified analyses of the association between *H. Pylori *and pancreatic cancer

The material was then stratified for smoking habits and alcohol consumption in order to study the association between a positive *H. pylori *serology and pancreatic cancer in subgroups defined by these risk factors (table 4). The size of the resulting subgroups was small and possible covariates were therefore entered one at a time. BMI and alcohol consumption measured by the Mm-MAST test were the two cofactors that had the most important impact on the association between positive *H. pylori *serology and pancreatic cancer in never smokers. The OR for pancreatic cancer related to positive *H. pylori *serology was 4.45 (1.19–16.69) when both these cofactors were entered in the analysis (not shown in table). *H. pylori *seropositivity was associated with the risk for pancreatic cancer in subjects with a low risk alcohol consumption in the unadjusted model (2.33 (1.09–4.97)). This association remained statistically significant when adjusting for all entered covariates, except for smoking status which resulted in a borderline significant result (2.13 (0.97–4.69)). In the small subgroup of subjects who reported a low risk alcohol consumption and were never smokers (8 cases and 55 controls), the crude OR for pancreatic cancer related to a positive *H. pylori *serology was 13.20 (2.31–75.31) (not shown in table).

**Table 4:** Odds ratios for pancreatic cancer with 95% confidence intervals by *Helicobacter pylori *serology stratified for smoking status and alcohol consumption (no reference categories shown in table)

Factors	Smoking status						Alcohol consumption **					
		
	Never smoker		Current smoker		Former smoker		Low risk		Intermediate risk		High risk	
						
	Cont	Case*	Cont	Case*	Cont	Case*	Cont	Case*	Cont	Case*	Cont	Case*
*H. pylori positive (n)*	*25*	*7*	*50*	*24*	*25*	*8*	*47*	*20*	*41*	*17*	*7*	*2*
*H. pylori negative (n)*	*63*	*6*	*65*	*31*	*35*	*11*	*82*	*15*	*72*	*25*	*7*	*5*
*H. pylori *(crude)	2.94 (0.90–9.61)		1.01 (0.53–1.92)		1.02 (0.36–2.90)		2.33 (1.09–4.97)		1.19 (0.58–2.47)		0.40 (0.057–2.80)	
*H. pylori *+ age	2.95 (0.89–9.81)		1.01 (0.53–1.92)		1.01 (0.35–2.87)		2.31 (1.08–4.97)		1.20 (0.58–2.48)		0.42 (0.058–3.04)	
*H. pylori *+ sex	3.04 (0.91–10.18)		1.01 (0.53–1.93)		0.99 (0.35–2.86)		2.33 (1.09–4.98)		1.23 (0.59–2.55)		0.34 (0.048–2.46)	
*H. pylori *+ time to analysis	3.40 (0.99–11.72)		1.00 (0.52–1.91)		1.07 (0.37–3.08)		2.32 (1.08–4.96)		1.25 (0.60–2.61)		0.37 (0.052–2.69)	
*H. pylori *+ BMI***	3.77 (1.05–13.48)		1.00 (0.52–1.92)		1.20 (0.41–3.53)		2.33 (1.09–4.97)		1.19 (0.57–2.46)		0.30 (0.035–2.61)	
*H. pylori *+ alcohol**	3.81 (1.06–13.63)		1.03 (0.53–1.99)		1.19 (0.39–3.61)		-		-		-	
*H. pylori *+ smoking status	-		-		-		2.13 (0.97–4.69)		1.29 (0.61–2.73)		0.29 (0.035–2.40)	
*H. pylori *+ all covariates	4.97 (1.23–20.10)		1.01 (0.52–1.97)		1.52 (0.45–5.10)		2.19 (0.98–4.88)		1.38 (0.64–2.98)		0.24 (0.023–2.48)	

*Controls/Cases

**Alcohol consumption estimated by the Malmö modification of the Michigan Alcoholism Screening Test. OR in subjects with missing alcohol consumption data are not reported due to small number of controls (n = 7) and cases (n = 3).

***BMI, body mass index

As a complement to the Mm-MAST test, γ-GT-values were used to provide an alternative marker for alcohol consumption. In subjects with a γ-GT value in the lowest tertile, the crude OR for pancreatic cancer for subjects with a positive compared to a negative *H. pylori *serology was 1.72 (0.75–3.96), in the middle γ-GT tertile it was 1.54 (0.65–3.66) and in the upper γ-GT tertile it was 0.90 (0.38–2.16) (not shown in table). In subjects who were both never smokers and presented at baseline investigation with γ-GT values in the lowest tertile, the crude OR for pancreatic cancer was 3.78 (0.79–18.13) for *H. pylori *positive vs. *H. pylori *negative subjects (not shown in table). Stratifying for BMI categories did not reveal any statistically significant association between *H. pylori *positive subjects and pancreatic cancer in any BMI category (data not shown).

## Discussion

The association between pre-diagnostic measurements of *H. pylori *serology and pancreatic cancer was investigated in this population-based cohort study including both men and women. *H. pylori *seropositivity was not associated with an increased risk for pancreatic cancer in the overall analysis. However, in never smokers, there was a statistically significant association between *H. pylori *and pancreatic cancer, and in subjects with low alcohol consumption there were also indications for such an association, although not statistically significant.

The validity of data collected at baseline investigation invites discussion. A previous study in the Malmö Preventive Medicine cohort has determined cut-off level of the ELISA and demonstrated that the immunoreactivity in the stored samples is stable despite the long storage time [7]. The prevalence of *H. pylori *seropositivity increases with age [18]. This is thought to be a birth cohort effect, ie the higher prevalence of seropositivity among elderly people reflects a higher childhood infection rate at the time when they were children rather than acquisition during adult life [19,20]. It is therefore reasonable to assume that most subjects with a negative *H. pylori *serology at baseline investigation probably remained uninfected until end of follow-up. Since controls were matched both for age and time of baseline investigation we do not believe that the changing *H. pylori *prevalence over time and age in this population has introduced any major bias of the results. It is possible that some of the *H. pylori *positive subjects have been eradicated after baseline investigation which may have attenuated a potential association between *H. pylori *infection and pancreatic cancer to some extent. However, since the baseline investigation in this study was performed in middle aged subjects, it is reasonable to assume that even an individual that was eradicated after the screening visit would have had a fairly long life-time exposure for *H. pylori *infection. Alcohol consumption was estimated by two separate means, the Mm-MAST test and γ-GT levels in serum. The Mm-MAST test is a validated questionnaire for detection of high risk alcohol consumption. The questionnaire is directed towards drinking behavior but does not contain questions on amounts of ingested alcohol [16]. It has been argued that leaving out this type of quantifying question would make the test a more valid tool for detecting individuals with a high risk alcohol consumption [16]. The validity of γ-GT as a tool for detection high alcohol consumption has been investigated in numerous previous studies. In the primary health care setting, the reported sensitivity is 20–40% and the specificity around 90% in most studies [21]. Using γ-GT instead of the Mm-MAST test to adjust for alcohol consumption in the logistic regression model gave similar results. The validity of self-reported data on smoking habits has been investigated previously in this cohort. A high agreement between plasma levels of carboxyhaemoglobin and self-reported smoking status was reported indicating that the risk for misclassification bias in this regard is probably low [22].

The ascertainment of cases is another potential source of misclassification bias. The Swedish Cancer registry has previously been reported to be 98% complete, indicating a low risk for missed cases [23]. In this study, all case files and pathology reports were reviewed in order to validate the diagnosis of pancreatic adenocarcinoma. The median age at diagnosis in our study was 60.7 years which is slightly lower than the median of 65 years that is usually reported for pancreatic cancer [2]. This can be explained by the fact that the majority of the cohort has not yet been followed to a very high age. Consequently, the results might not be applicable to pancreatic cancer occurring at higher ages. It is possible that participants and non-participants in the Malmö Preventive Project differed regarding the distribution of risk factors or the type of pancreatic cancer. However, the high attendance rate (71%) and the population-based recruitment are considerable strengths in this study that have probably limited a potential selection bias.

The present study included 87 cases which makes it slightly smaller than the previously published studies from Finland and the US [11,12]. Hence, it is possible that the lack of a statistically significant association between *H. pylori *serology and pancreatic cancer in the overall analysis and among current smokers is due to a type II error. That is, we may have missed a true difference due to poor statistical power. However, it is probable that such a potential association is not very strong, considering that the estimated OR with 95% CI for pancreatic cancer among *H. pylori *positive subjects was 1.25 (0.75–2.09) in the total cohort and 1.01 (0.53–1.92) among current smokers in this study. Furthermore, our results are in accordance with the US study [12].

The association between *H. pylori *seropositivity and pancreatic cancer in both never smokers and low risk consumers of alcohol is a potentially important observation but being a subanalysis it has to be interpreted cautiously. These subgroups were small and, as a consequence, it was difficult to adjust for all potential confounders on the same time in the statistical model. However, the inclusion of these variables one at a time did not influence the results to any major extent, and we consider that confounding due to these factors can only have been a minor problem.

The association between *H. pylori *and pancreatic cancer has been investigated in three previous studies. A case control study was performed in Austria by Raderer *et al *on 92 patients with pancreatic cancer that were compared to a control group consisting of 35 patients with colorectal cancer and 27 healthy volunteers [10]. *H. pylori *seropositivity was associated with an OR of 2.1 (1.1–4.1) for pancreatic cancer in that study. A nested case-control study on the association between *H. pylori *serology and pancreatic cancer in a Finnish cohort was performed by Stolzenberg-Solomon *et al *[11]. In that study the OR for pancreatic cancer was 1.87 (1.05–3.34) adjusted for total time of smoking. Recently, a nested case-control study on the association between *H. pylori *and pancreatic cancer within a US cohort of subscribers to the Kaiser Permanente Medical Care Program, including 104 cases and 262 controls, was published [12]. In contrast to the previous two studies, no association between *H. pylori *infection and pancreatic cancer was observed. Subgroup analysis did not reveal any association in smoking men. The association between *H. pylori *serology and pancreatic cancer among never smokers was not reported. The relatively large sample size and the nested case-control design with analysis of *H. pylori *serology on prospectively collected blood samples are strengths in the latter two studies. However, it may be difficult to compare our results to the Finnish study since it only included smoking middle-aged men. Our findings are in accordance with the US study that was performed in a more heterogeneous cohort including both men and women regardless of smoking habits. A strong point in our study is that it is performed in a population based cohort, facilitating generalization of the results to other populations.

Pancreatic secretion is under hormonal control mainly by cholecystokinin which stimulates enzyme production and secretion from acinar cells and by secretin which induces bicarbonate secretion from ductal cells [24]. Secretin is released by cells in the duodenal wall in response to a local fall in pH that occurs when foods mixed with gastric acid enters the duodenum [25]. Several mechanisms have been proposed for the potential association between *H. pylori *infection and pancreatic cancer. Antral colonization by *H. pylori *has been associated with increased gastric acid output which will lead to increased secretin release from the duodenum. Secretin stimulation has been proven to accelerate the development and frequency of pancreatic tumors induced by nitrosamines in a hamster model [26]. One possible hypothesis is that the increased secretin levels associated with antral *H. pylori *infection, either per se or by acting as a cocarcinogen, increase the risk of pancreatic cancer [27]. Opposite to antral colonalization, *H. pylori *infection of the corpus area of the stomach is associated with a loss of parietal cells and a decrease in gastric acid out-put [27]. A second mechanism for an association between *H. pylori *infection and pancreatic cancer has been proposed derived from this model. Hypoacidity can lead to bacterial overgrowth and increased production of N-nitroso compounds which can be activated in the ductal epithelium after transportation to the pancreas by the circulation. This second hypothesis related to hypoacidity is supported by the observation that pernicious anemia is associated with pancreatic cancer [28,29]. An increased risk for pancreatic cancer was demonstrated in patients with gastric, but not duodenal ulcers in a recently published register based Swedish study by Luo *et al *[30]. This observation suggests that if there is an association between *H. pylori *and pancreatic cancer, the second hypothesis with decreased acid out-put due to *H. pylori *infection of the gastric mucosa is the probable mechanism since gastric, but not duodenal, ulcers are associated with this form of infection.

The possibility of intrapancreatic invasion by *Helicobacter *species has been investigated in resections specimens from pancreatic cancer, chronic pancreatitis and normal pancreatic tissue [31]. In that study *Helicobacter *DNA could be detected in 30 out of 40 patients with pancreatic cancer, and in 3 out of 5 patients with chronic pancreatitis. All 7 samples from normal pancreas were *Helicobacter *negative. Increased secretion of vascular endothelial growth factor and interleukin 8 has been observed in vitro after incubation of pancreatic cancer cell-lines with H pylori, providing a possible way for how H pylori could increase the malignant potential if intrapancreatic infection occurred [32]. The relevance of these findings remains to be elucidated.

Gastric acid secretion has been demonstrated to be influenced by smoking [33-35] and consumption of non-distilled alcoholic beverages produced by fermentation has been proven to increase gastric acid out-put and gastrin release [36]. Moreover, smoking is an important source of n-nitroso compound exposure in humans [37], and increased concentrations of tobacco-specific nitrosamines has been demonstrated in the pancreatic juice of smokers [38]. In the present study, there was an association between *H. pylori *seropositivity and pancreatic cancer only in never smokers and in subjects with low risk alcohol consumption. A possible explanation to this is observation could be that the effect of a weak risk factor such as *H. pylori *infection is more important in the absence of stronger risk factors such as smoking and alcohol consumption since all three possible risk factors have been suggested to influence the risk for pancreatic cancer through the same mechanisms, i.e. gastric acid secretion and production of n-nitroso compounds.

## Conclusion

In summary, we did not find any statistically significantly increased risk for pancreatic cancer among subjects with a positive *H. pylori *serology in the overall analysis in this nested case-control study. We cannot completely exclude that the lack of an association between H. Pylori seropositivity and pancreatic cancer in our study was due to insufficient statistical power but our results indicate that *H. pylori *infection is probably not a strong risk factor for pancreatic cancer in the general population. However, a positive *H. pylori *serology was associated with an increased risk for pancreatic cancer in subgroups of subjects classified as never smokers or low risk consumers of alcohol. These observations should be interpreted cautiously, bearing in mind the limited number of cases in these subgroups.

## Competing interests

The authors declare that they have no competing interests.

## Authors' contributions

BL contributed to the planning of the study, performed the data analyses and drafted the manuscript. DJ contributed to the planning of the study, validated all cases of pancreatic cancer, took part in the interpretation of the results, reviewed the manuscript, and approved the final version. AB was responsible for the planning of the study and obtained funding. JM obtained funding, supervised data analysis, interpretation of the results and manuscript writing and approved the final manuscript.

## Note

† The author's wish, with appreciation, to acknowledge the contribution made to this paper by Professor Anders Borgström, who died on 9th October 2007.

## Pre-publication history

The pre-publication history for this paper can be accessed here:


